# Novel Aminoacridine Sensors Based on Molecularly Imprinted Hybrid Polymeric Membranes for Static and Hydrodynamic Drug Quality Control Monitoring

**DOI:** 10.3390/ma12203327

**Published:** 2019-10-12

**Authors:** Saad S. M. Hassan, Abd El-Galil E. Amr, Heba Abd El-Naby, Mohamed El-Naggar, Ayman H. Kamel, Nagy M. Khalifa

**Affiliations:** 1 Department of Chemistry, Faculty of Science, Ain Shams University, Abbasia 11566, Cairo, Egypt; hoba_science@hotmail.com; 2 Pharmaceutical Chemistry Department, Drug Exploration & Development Chair (DEDC), College of Pharmacy, King Saud University, Riyadh 11451, Saudi Arabia; nkhalifa.c@ksu.edu.sa; 3 Applied Organic Chemistry Department, National Research Center, Dokki 12622, Giza, Egypt; 4 Chemistry Department, Faculty of Sciences, University of Sharjah, Sharjah 27272, UAE; m5elnaggar@yahoo.com

**Keywords:** potentiometric sensors, aminoacridine, quality control, flow injection analysis (FIA)

## Abstract

Novel biomimetic potentiometric ion-selective electrodes (ISEs) were fabricated and designed for the assessment of aminoacridine (ACR) based on newly synthesized imprinted polymer (MIP) membranes. Thermal polymerization of methacrylic acid (MAA) or acrylamide (AM) as function monomer, aminoacridine as a template and ethylene glycol dimethacrylate (EGDMA) as across-linker, were utilizedto give the molecular recognition part. The membranes of sensors I andII consist of MIP based MAA and AM, respectively, dispersed in a poly(vinyl chloride) membrane plasticized with dioctyl phthalate (DOP) in the ratio of 3.0 wt%, 32.2 wt% and 64.8 wt%, respectively. Sensors III and IV were similarly prepared with added 1.0 wt% tetraphenyl borate (TPB^−^) as an anionic discriminator. Sensors I and II exhibited near-Nernstian potential response to ACR^+^ with slopes of 51.2 ± 1.3 and 50.5 ± 1.4 mV/decade in a 0.01 M phosphate buffer of pH 6.0. The linear response coversthe concentration range of 5.2 × 10^−6^ to 1.0 × 10^−3^ M with a detection limit of 0.05 and 0.17 μg/mL for sensors I and II, respectively. The performance characteristics of these sensors were evaluated under static and hydrodynamic mode of operations. They were used for quality control assessment of aminoacridine in some pharmaceutical preparations and biological samples.

## 1. Introduction

Acridine and its derivatives are antimicrobial agents, effective against a wide range of microorganisms. Aminoacridine is commonly used in the treatment of infections of vaginal candidacies, trichomonavaginitis, haemophilia, moniliasis, and as prophylactic agent in various gynaecological procedures [1]. 9-Aminoacridine is the most active among all aminoacridine derivatives due to its higher basicity. It is used also in the treatment of tinea versicolor and mastitis [2]. It is also used as a safe and effective irrigant in dentistry. Available literature confirms that it is a potent antimicrobial agent, effective against a wide range of microorganisms commonly found in septic wounds and causing minimal tissue irritation [3]. In addition, it is used in dentistry for surgical root canal irrigation and as antiseptic in management of maxillofacial abscesses [4]. However, few methods are available in the literature for the determination of acridine and its derivatives. Chemilumiscence measurement after oxidation with permanganate and optical solvatochromic method has been suggested [5]. Non-aqueous potentiometric titration with perchloric acid or titration with chlorosulphonic and location of the end point by potentiometry and photometry has been described [6,7]. Adsorptive stripping voltammetry at a static mercury dropping electrode has been used for acridine assessment [8]. Reversed phase HPLC, TLC and GLC have been utilized for separation and quantitation of aminoacridine [9,10] and spectrophotometric and fluorimetric methods for aminoacridine determination [11,12]. Most of these methods suffer from lack of selectivity and sensitivity or involved several time consuming manipulation steps. Although potentiometric polymeric membrane ion selective sensors find wide applications in environmental, clinical and pharmaceutical analysis due to simplicity, accuracy and selectivity, no single sensor has been described for determination of acridine compounds.

Molecular imprinting technology had increased interest in the recent years [13]. The importance of this technique was reflected in several reviews [14,15,16,17]. They discussed the application of MIPs in different fields such as solid phase extraction, chromatographic separations or as drug delivery systems [14,15,16,17]. Regarding chemical sensing, Haupt and Mosbach [15], presented a review about MIPs and their use in biomimetic sensors. On the other hand, molecularly imprinted technology (MIT) is one of the most promising methodologies for the conjunction with potentiometric sensors. They have been successfully developed for the assessment of various analytes [18,19,20,21,22]. In this respect, achievement of improved selectivity may be done by means of using MIP as a sensing element. In addition, they have a pre-defined specific cavities designated for the desired analyte, stable to wide pH range, organic solvents and temperature variations. These features provide for high flexibility in analytical and bio-analytical methods development [23,24,25,26].

In this work, novel molecularly imprinted 9-aminoacridine (ACR) polymers were prepared, characterized, incorporated in a poly(vinyl chloride) matrix membranes and utilized in conjunction with a potentiometric transduction for selective determination of low concentration levels of 9-aminoacridine in drug formulation and biological matrices.

## 2. Materials and Methods

### 2.1. Apparatus and Reagents

The potential measurements were conducted at 25 ± 1 °C with an Orion (Cambridge, MA, USA) Model 720 /SA pH /mV meter using molecularly imprinted aminoacridine membrane sensor in conjunction with an Orion Ag/AgCl double-junction reference electrode (Model 90-20) filled with 10% (w/v) KNO_3_. All pH measurements and solution pH adjustments were made with a combination Ross glass electrode (Orion 81-02).Continuous analysis was performed using a flow injection manifold consisted of a two-channel peristaltic pump (Ismatech Ms-REGLO), polyethylene tubing (0.71 mm i.d.) and an Omnifit injection valve (Omnifit, Cambridge, UK) with a sample loop of 100 μL volume. The potential signals were recorded using data acquisition (eight-channel electrode-computer interface (Nico- 2000 Ltd., London, UK) controlled by Nico-2000 software).

All chemicals were of analytical grade or the highest purity available. 9-Aminoacridine (ACR) from National Authority for Control and Pharmaceutical Research (Egypt), high molecular weight poly (vinyl chloride) (PVC), Dioctylphthalate (DOP) acrylamide (AM), methacrylic acid (MAA) and ethylene glycol dimethacrylate (EGDMA) were used as received from Fluka (Ronkonoma, NY, USA). Phosphoric acid, and tetrahydrofurane (THF), were purchased from Sigma (St. Louis, MO).Benzoyl peroxide (BPO) from Riedel-deHaen. Methanol, acetic acid and acetonitril were bought from Merck. All measurements were done in 1.0 × 10^−2^ M phosphate buffer at pH 6.0. A stock solution of 1.0 × 10^−2^ M ACR was prepared in distilled water. Working solutions (1.0 × 10^−3^–1.0 × 10^−6^ M) were prepared by accurate dilutions and stored in brown bottles.

### 2.2. Synthesis of Molecularly Imprinted Polymers

MIP beads were prepared through the non-covalent approach [27]. Briefly, the template (ACR, 0.4 mmol), methacrylic acid or acrylamide (MAA or AM, 5 mmol), ethylene glycol dimethacrylate (EGDMA, 24 mmol) and benzoylperoxide (BPO, 50 mg) were dissolved in acetonitril (AN, 10 mL) in a 50 mL flask. The mixture was sonicated for 5 min to achieve homogeneity then degassed by N_2_ for 10 min. The polymerization was performed at 70 °C in a water bath for 18 h. After polymerization, the template was removed by batch-mode solvent extraction using methanol/acetic acid (8:2) and methanol. The resulting polymer was dried in vacuum overnight at room temperature. The non-imprinted polymer (NIP) particles were prepared by the same procedure in absence of the template. 

### 2.3. Membrane Sensors and Potential Measurements

The membranes of the sensors used for batch measurement of the drug were prepared by dissolving 570 mg of the components [MIP or NIP (30 mg), PVC (190 mg), and DOP (350 mg)] in 5 mL THF in a (5 cm diameter) glass ring. The membranes of sensors (I) and (II) consist of MIP based MAA and AM, respectively. Sensors (III) and (IV) were similarly prepared with added 1.0 wt% TPB as an anionic discriminator.Membranes containing NIP based MAA and AM were also prepared to make sensors (V) and (VI), respectively. All membrane solutions were left to stand overnight at room temperature to evaporate the solvent slowly. The resulting membrane was peeled off from the glass ring and discs of 9 mm i.d. were cut out and glued onto a 7-mm i.d PVC body using THF. The tube was filled with 10^−2^ M aminoacridinium hydrochloride as internal solution and 3 mm diameter Ag/AgCl coated wire was used as an internal reference electrode. A 1.0 × 10^−2^ M aqueous ACR solution was used as a conditioning solution for sensors, and stored in the same solution when not in use. 

The potential measurements under a static mode of operation were recorded after immersion of the sensors in conjunction with double-junction Ag/AgCl reference electrode in solutions containing a 1.0 × 10^−2^–1.0 × 10^−6^ M of ACR. All solutions were adjusted at pH 6.0 with 0.01 M phosphate buffer. The potential readings were recorded after stabilization to ±0.5 mV and the EMF was plotted as a function of the logarithm (ACR) concentration. The life time of the membranes was 2 months.

**Figure 1:**
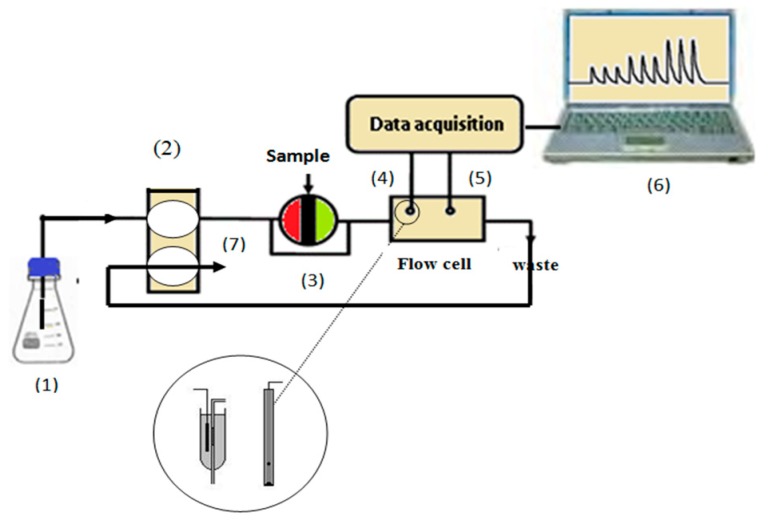
Schematic diagram of the flow injection system. (1) 10^−2^ M phosphate buffer carrier; (2) peristaltic pump; (3) loop sample 100 μL; (4) working electrode; (5) reference electrode; (6) laptop; (7) waste; conditions: flow rate 3.5 mL/min.

The detector used for continuous drug analysis was prepared as described previously [28]. The membrane cocktail was deposited; using a micro-dropper, three to four times in a smallhole (3 mm wide × 5 mm length) made in the middle of a 15 cm Tygon tube (0.071 i.d.). The tube was inserted and sealed with Araldite in a 100 μL pipette tip (7 cm long, 0.4 cm diameter). The formed membrane sensor was inserted into the flow injection system as schematically shown in Figure 1. The end of the tube was placed in a Petri dish where a double junction Ag/AgCl reference electrode was placed down-stream from the indicator sensor just before the solution went to waste. The sample loop (100 μL) of the injection valve was filled and the valve was rotated to allow the sample to be carried out by 0.01 M phosphate buffer stream of pH 6.0 to the flow-through cell. The potential signals were recorded using data acquisition (eight-channel electrode-computer interface (Nico-2000 Ltd., London, UK) controlled by Nico-2000 software). Each sample was measured in triplicate runs and the average peak height was recorded and compared with a calibration plot under the same condition.

### 2.4. Binding Capacity of Aminoacridine Molecularly Imprinted Polymer

All polymers were analyzed for binding template using equilibrium binding experiments [29]. This was carried out by placing (30 mg) of MIP or NIP particles in contact with 10 mL of ACR solutions ranging from 0.01–10 mM. The solutions were incubated overnight for a static equilibrium at room temperature and centrifuged at 6000 rpm for 15 min. The solid phase was then separated and the free ACR concentration was measured by sensor (II).

### 2.5. Selectivity Towards Other Cations

The potentiometric selectivity coefficients (*K^pot^_i,j_*) of the proposed ACR sensors towards different cationic species (*j*) were measured using the fixed interference method (FIM) [30]. A 1.0 mL aliquot of 1 × 10^−2^ M of the interfering ion (*j*) was transferred into a 50 mL beaker containing 9.0 mL of phosphate buffer of pH 6.0 and the sensor in conjunction with a double junction Ag/AgCl reference electrode was immersed in the solution and calibrated with ACR stock solution. The selectivity coefficient was measured and calculated from the rearranged Nicolsky equation [30]:

(1) $$\log K_{i,j}^{pot}\;=-\;\log\;a_{j}^{\left(Z_{i^{}}/Z_{j}\right)}\;+\log a_{i}$$

where *a_i_* = *a_j_* = 10^−3^ M are the activities and *z_i_* and *z_j_* are the charges of ACR and the interfering ion respectively. The selectivity of sensors (III) and (IV) where tetraphenyl borate (TPB) was used as cationic additive was also studied.

### 2.6. Determination of ACR in Biological Fluids

To test the applicability of the sensors in more complicated matrices, known addition (spiking) technique was followed and the concentration of ACR in different plasma and urine samples was measured under both static and hydrodynamic mode of operation. A 10 mL aliquot of plasma or urine sample was transferred, without removal of any particulate matter, to a 100 mL measuring flask and diluted to the mark with 0.01 M phosphate buffer solution of pH 6.0. A 1.0 mL aliquot was transferred to 25 mL beaker containing 9 mL buffer solution. Portions (1.0 mL) of standards 1.0 × 10^−4^, 5.0 × 10^−4^, 1.0 × 10^−3^ and 1.0 × 10^−2^ M ACR representing samples (S_1_, S_2_, S_3_, S_4_) were added to the buffered urine solution. Portions (1.0 mL) of the standard 1.0 × 10^−4^, 1.0 × 10^−3^ and 1.0 × 10^−2^ M ACR representing samples (S_5_, S_6_, S_7_) were added to the buffered plasma solution. Each of these solutions was thoroughly mixed and the aminoacridine sensor in conjunction with the reference electrode was immersed in the solution. The potential readings were recorded after reaching the equilibrium response (10–20 s). 

### 2.7. Determination of ACR in Pharmaceutical Samples

An accurately weighed 40 g of Septgel drug containing 0.01/20 (*w*/*w*) aminoacridine was transferred into a 100 mL volumetric flask. 0.01 M phosphate buffer of pH 6 was added to dissolve and dilute the sample (S_8_) to the flask mark. The solution was measured as described above and the potential reading was compared with a calibration plot under similar conditions under both static and hydrodynamic modes of operation.

## 3. Results and Discussions

### 3.1. Characterization of the MIP Particles

As artificial mimetic, MIPs have proven to be excellent receptors for the selective recognition of organic targets in chemical sensors [11,12,18,19,20,22,23,24]. In this work we explore for the first time the feasibility for fabricating a potentiometric sensor based on MIP in the polymeric membrane as a selective receptor for 9-aminoacridine. In the polymerization step, MAA and AM were tested as functional monomers in presence of EGDMA as a cross-linker. The carboxylic group (COOH) of MAA or amide group (–CONH_2_) of AM can interact through strong hydrogen bonding with the amino (–NH_2_) and basic nitrogen in the pyridine ring. In addition, contribution of π-interactions from the aromatic moiety of the template towards the electron deficient centers in the polymer structure is considered. Formation of aminoacridine MIPs were confirmed by the Fourier-transform-infrared spectroscopy (FT-IR), and characterized by using scanning electron microscopy (SEM). 

**Figure 2:**
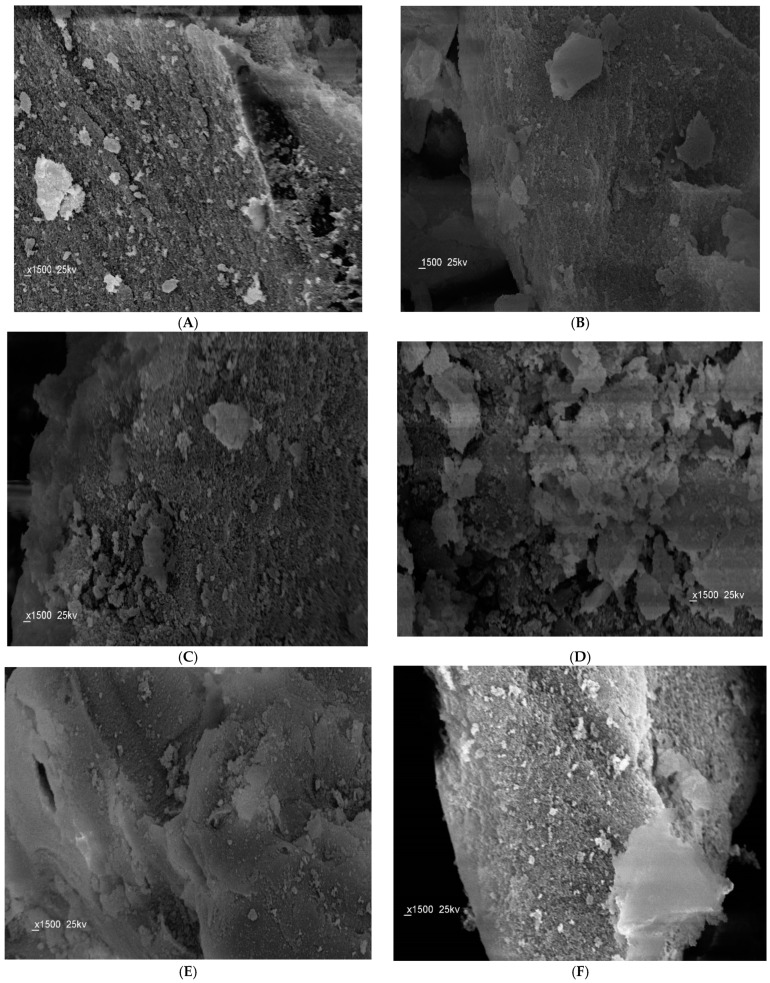
SEM images of the obtained MIP and NIP beads: (**A**) non-washed MAA/MIP; (**B**) washed MAA/MIP; (**C**) MAA/NIP; (**D**) non-washed AM/MIP; (**E**) washed AM/MIP, and; (**F**) AM/NIP.

The SEM images of the prepared polymeric materials were shown in Figure 2. The different surface structures can come from different physical properties during polymerization. The presence of the template during polymerization can cause different particles even if there’s no imprinting effect.All the beads of MIP have an irregular and rough surface due to the high amount of the cross-linker added during the polymerization process and to the pores formed during the imprinting of ACR on the polymers. For NIP beads, they have more smooth and uniform shapes than MIPs because ACR wasn’t entered in the polymerization process. The diameter distribution of the micro-beads was 2.1–2.4 μm and 1.23–1.54 μm for MIP and NIP particles, respectively. This difference between the size of MIP and NIP particles can be attributed to the imprinting effect.

**Figure 3:**
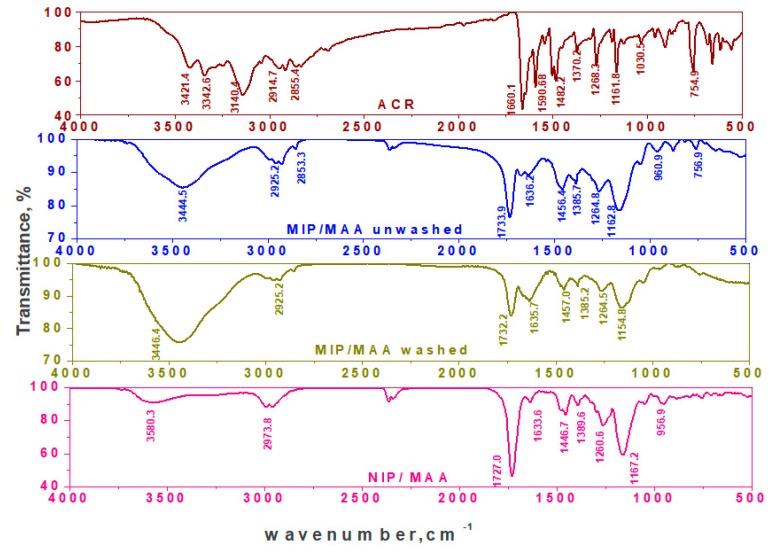
FTIR spectra of ACR, MIP with MAA monomer and NIP.

**Figure 4:**
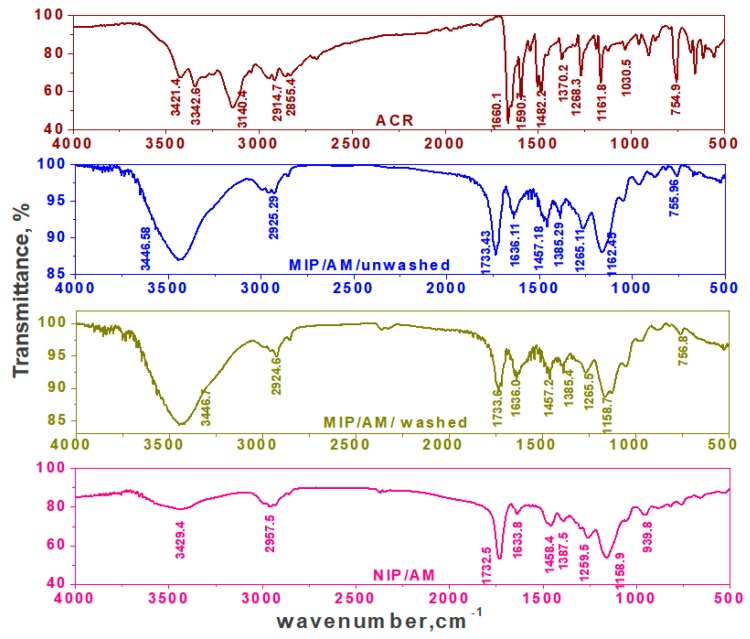
FTIR spectra of ACR, MIP with AM monomer and NIP.

The FT-IR spectra of MIPs particles confirm the imprinting process through the absence and presence of ACR on the surface of both washed or non-washed polymer particles, respectively Figure 3 and Figure 4. Nicolet™ iS50 (ATR-FTIR) Spectrometer in a spectral range of 4000–500 cm^−1^ was used. From the FT-IR analysis, it can be seen that ACR is characterized by the presence of bands due to γ_N–H_ (stretching) at 3421, 3342 cm^−1^, γ_N–H_ (bending) at 1590 cm^−1^, γ_C–H_ (stretching, aromatic) at 2914, 2855 cm^−1^, sharp and strong stretching γ_C=C_ (stretching) at 1660 cm^−1^, and γ_C–N_ (stretching) in the aromatic ring and C-N at position (9) at 1268 and 1161 cm^−1^, respectively. The FT-IR spectrum of non-washed MIP based on MAA as a functional monomer showed O–H stretch due to methacrylic monomer at 3444 cm^−1^. The band due to γ_N–H_ (bending) at 1590 cm^−1^ was disappeared and that due to γ_C–N_ band at 1162 cm^−1^ was broadened. This confirms that the imprinted process proceeded through the hydrogen bond between the amino group of the template and the amide group in MAA monomer. In addition, there was a shift of γ_C=O_ (stretching) of the amide group from the cross linker to 1733 cm^−1^ and that γ_C=O_ (stretching) of the amide group of the monomer to 1636 cm^−1^. The two peaks at about 1727 and 1167 cm^−1^, corresponding to –C=O or –C–O stretches, respectively, are common in all spectra because of the EGDMA cross-linker used. For NIP/MAA/ACR, it was characterized by γ_N–H_ (stretching) band at 3580 cm^−1^, strong and sharp γ_C–O_ (stretching) band at 1167 cm^−1^ and γ_C–H_ (stretching) band at 2973 cm^−1^ that fairly agreed with those obtained for MIP/MAA/ACR after template removal. The FT-IR spectrum of non-washed MIP based on AM as a functional monomer showed that, the γ_N–H_ (stretching) was shifted to 3446 cm^−1^ with more broadening, disappearance of γ_N–H_ (bending) located at 1590 cm^−1^ and broadening in γ_C–N_ band located at 1161 cm^−1^. This confirms that the imprinted process proceeded through the hydrogen bond between amino group in the template and the amide group in AM monomer. In addition, there was a shift for γ_C=O_ (stretching) of the amide group from the cross linker to 1733 cm^−1^ and that γ_C=O_ (stretching) of the amide group from the monomer shifted to 1636 cm^−1^. The two peaks at about 1732 and 1158 cm^−1^, corresponding to–C=O or –C–O stretches, respectively, are common in all spectra because of the EGDMA cross-linker used. For NIP/AM/ACR, it was characterized by γ_N–H_ (stretching) band at 3429 cm^−1^, strong and sharp γ_C-O_ (stretching) band at 1158 cm^−1^ and γ_C–H_ (stretching) band at 2957 cm^−1^. These bands are similar to those obtained with MIP/AM/ACR after template removal. The coincidence of the spectrum of NIP beads and that of washed MIPs, confirms the successful removal of the template after washing.

### 3.2. Binding Capacity of MIPs and Scatchard Analysis

**Figure 5:**
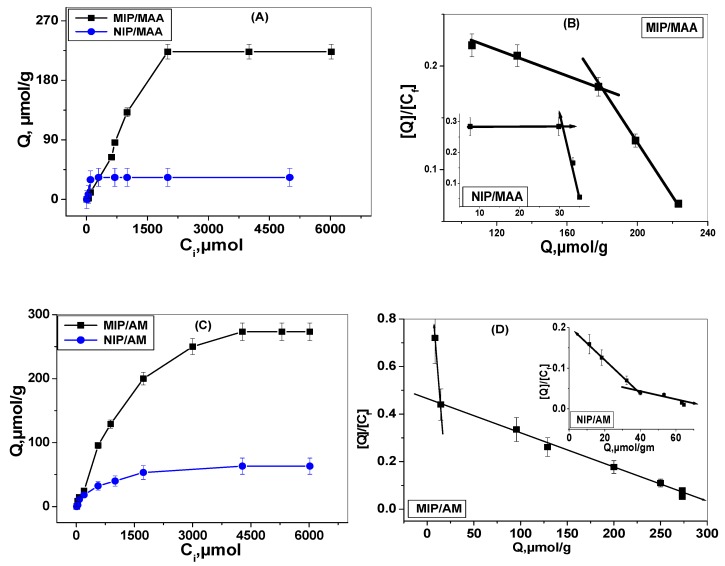
Binding isotherm and Scatchard analysis for (**A**) MIP/MAA, (**B**) NIP/MAA, (**C**) MIP/AM and (**D**) NIP/AM polymers, Conditions: 30.0 mg of the respective polymer; *t* = 25 °C; V = 10.0 mL; binding time: 24 h.

The capacity of MIPs up take ACR was investigated using equilibrium binding experiments [29]. The mode of binding, site distributions in the interaction and adsorption isotherms were examined by using a fixed amount (30 mg) of MIP or NIP beads and varying initial concentrations (0.01 to 10 mM) of the ACR. After soaking for 24 h at ambient temperature the capacity of MIPs was calculated according to following equation:

(2) $$Q=\frac{mmol\;\left(ACRbound\;\right)}{g\left(MIP\right)}\;=\frac{\left(C_{i}-C_{f}\right)V_{s}\times 1000}{M_{MIP}}$$

where *Q* is binding capacity of MIP or NIP (mmol g^−1^), *C_i_* the initial ACR concentration (mmol mL^−1^), *C_f_* the final ACR concentration (mmol mL^−1^), *V_s_* the volume of solution tested (mL) and *M_MIP_* the mass of dried polymer (mg). The adsorption isotherms for MIPs were shown in Figure 5A,C. It can be seen from the curves that the binding amounts for MIP and NIP polymers increase with increasing the amount of ACR concentration. At the concentration of 5 mM ACR, the bounded amount of ACR by the imprinted polymer is 273 and 223.3 μmol g^−1^ for MIP/AM and MIP/MAA, respectively. For non-imprinted polymers at the same ACR concentration, the bounded amount by the polymer is 63.3 and 33.4 μmol g^−1^ for NIP/AM and NIP/MAA, respectively.

The Scatchard Model was often used to evaluate the binding characteristics of the imprinted polymers through the following equation [31]:

[*Q*]/[*C_free_*] = ([*Q_max_*] − [*Q*])/*K_d_* (3)

where [*C_free_*] is the remaining concentration of the substrate in the supernatant, [*Q_max_*] is the maximum binding capacity of the binding site and *K_d_* is the equilibrium dissociation constant. *[Q]/[C_free_]* was plotted versus *Q* as shown in Figure 5B,D. These figures illustrate that the binding sites of MIP beads for ACR were heterogeneous with linear relationships which indicate the presence of two classes of binding sites with different affinities in the range of the different concentrations. The equilibrium dissociation constants *K_d1_, K_d2_* and the apparent maximum amount *Q_max1_*, *Q_max2_* for the higher and lower affinity binding sites are summarized in Table 1. These data showed that MIP/AM has a higher binding affinity and maximum binding capacity towards ACR than MIP/MAA.

### 3.3. Sensors Performances

**Figure 6:**
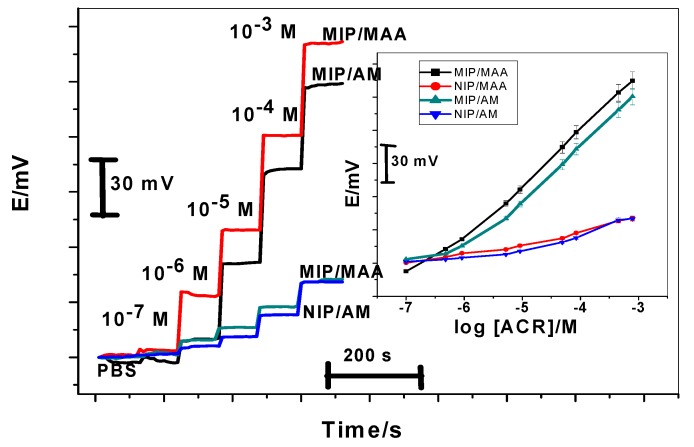
Response time and potentiometric response of aminoacridine (ACR) PVC membrane sensors based on molecularly imprinted polymers (MIPs) in 1 × 10^−2^ M phosphate buffer of pH 6.0.

The electrochemical characteristics of ACR membrane-based sensors were evaluated according to IUPAC recommendations [32]. Their general analytical features are presented in Table 2. It should be noted that Polymeric membrane sensor have the advantages of offering the possibility of controlling electrode characteristics such as hydrophobic/hydrophilic character, permeability and film thickness, all of which are essential for obtaining good sensor performance. The imprinting polymer is able to rebind again to its imprinted cavity after washing out the template, thus acting as a recognition element on a potentiometric sensors. In this work, a mimic receptor prepared by molecularly imprinting technique with (MAA or AM) as functional monomers and ethylene glycol dimethacrylate (EGDMA) as a cross linker, dispersed in PVC matrix plasticized with DOP solvent mediator was tested as a sensor for the determination of ACR. The sensors exhibit linear potentiometric response to ACR^+^ ion with slope of 51.2 ± 1.3 (*r*^2^ = 0.9993) and 50.5 ± 1.4 (*r*^2^ = 0.9999) mV decade^−1^ and detection limits of 2.5 × 10^−7^ and 8.7 × 10^−7^ M for sensors (I) and (II), respectively. Sensors (V) and (VI) based on NIPs exhibited no noticeable response. They revealed a potentiometric slope of 15.5 ± 1.1 and 16.3 ± 0.7 mV decade^−1^ over the linear range 10^−4^–10^−3^ M with detection limits of 6.0 × 10^−5^ and 6.5 × 10^−5^ M for sensors (V) and (VI), respectively. The potential response obtained with the sensors prepared with ACR-MIP or NIP membrane is given in Figure 6.

The influence of pH on the potentiometric response of the MIP based sensors was examined with standard 10^−4^ and 10^−3^ M ACR solutions over a pH range of 2–10. The pH of the solution was adjusted with either hydrochloric acid and/or sodium hydroxide solutions. The sensors exhibited good stability over the pH range 4.0–7.0 and 3.5–7.0 for sensors (I) and (II), respectively. In this ranges, the percentage of ionization exceeds 99.9% for ACR that behaved as monovalent cation and this can be attributed to its pKa (9.29) [33]. All subsequence measurements were carried out in 0.01 phosphate buffer at pH (6.0). The pH profile of the proposed sensors show that at pH ˃ 7.0 the potentials of the sensors declined with negative drift probably attributed to de-protonation of the drug molecules and formation of neutral aminoacridine species. 

From industrial point of view, stability of MIPs are crucial, therefore life time of membranes was studied in this work [34]. The time required to obtain a steady potential response (±2 mV) using the proposed sensors in 1.0 × 10^−7^–1.0 × 10^−3^ M ACR solutions with a rapid 10-fold increase in drug concentration were 10 s for both sensors. After several calibrations for each sensor, low potential drift, long-term stability and negligible change in sensors response were observed. When not in use, the sensors were stored and conditioned in 10^−2^ M ACR. All sensors examined exhibited good performance within reproducibility ±3% for calibration slopes. The linear range and detection limits over a period of at least 8 weeks are reasonably stable. The long-term stability of the proposed sensors was evaluated by testing the sensors in different ACR concentrations (10^−^^4^ and 10^−^^3^ M) for 8 weeks. The sensitivity (slope of the calibration curve) was calculated according to three assay results each day. The variation of the slope of calibration plots is less than 3.0 mV decade^−1^ over 60 d, thus showing a fairly good stability.

### 3.4. Sensors Selectivity

The selectivity behavior of ISEs is defined by the ion exchange constants which depend on the selectivity of complexation as well as on the standard free energies of the respective ions in the aqueous and organic phases [35]. Selectivity coefficients (Log *K^pot^_i,j_*) of the sensors were determined using fixed interference method (FIM) with 1.0 × 10^−3^ M of the interfering in 0.01 mol L^−1^ phosphate buffer at pH 6.0. Both sensors exhibite no interference towards a range of organic copmounds as shown in Table 3. Sensors (I) and (II) displayed the same selectivity for hydroxylamine, 3-aminopyridine and hexamine but sensor (II) was more better in the presence of histidine, alanine, piperidine, dimethylamin and methylaminecompared with sensor (I). On the other hand, sensor (III) with TPB displayed better selectivity than sensor (II) for all the tested interferences while sensor (IV) with TPB displayed no improvement in the selectivity behavior. 

### 3.5. Hydrodynamic Monitoring of Aminoacridine

**Figure 7:**
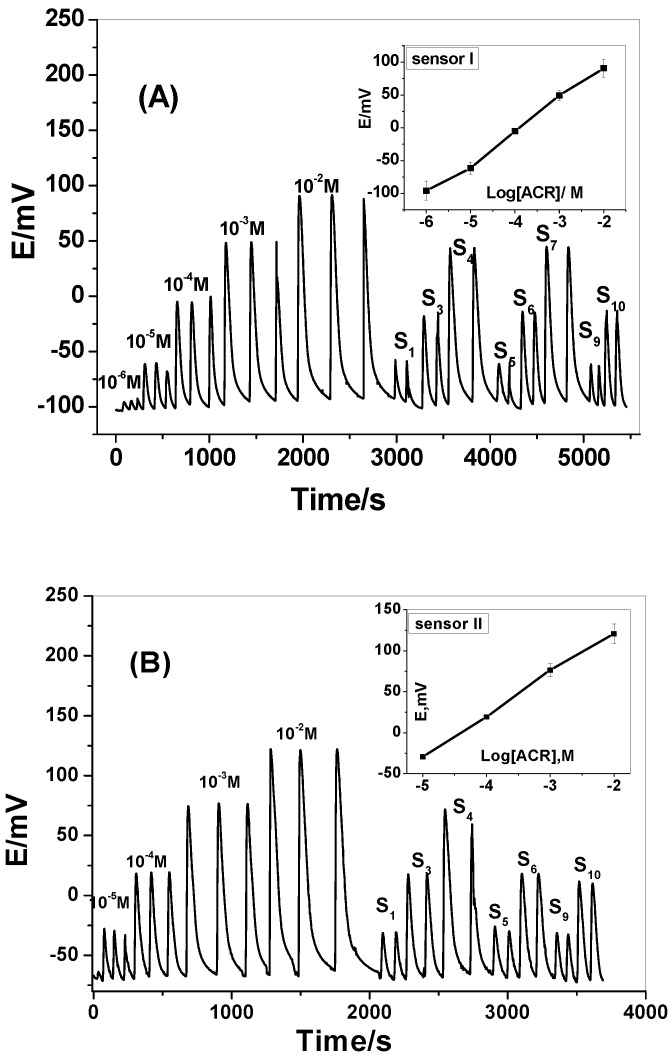
Transient potentiometric signals of MIP membrane based sensors plasticized with DOP. Conditions: carrier solution, 1 × 10^−2^ M phosphate buffer of pH 6.0; flow rate 3.5 mL/min; sample size 100 μL. (**A**) Sensor I; (**B**) Sensor II.

A tubular-type detectors incorporating MIP/MAA and/or MIP/AM based membrane sensors were prepared and used under hydrodynamic mode of operation for continuous ACR determination. A linear relationship between the ACR^+^ concentrations and FIA signals was obtained over a concentration range of 1.0 × 10^−5^ to 1.0 × 10^−2^ M using a 1.0 × 10^−2^ M phosphate buffer of pH 6.0 as a carrier solution and a flow rate of 3.5 mL/min. The general analytical features recorded under optimum flow conditions are presented in Table 4. The sensors exhibit linear potentiometric response to ACR^+^ ion with slopes of 51.0 ± 0.3 (*r*^2^ = 0.998) and 50.7 ± 0.2 (*r*^2^ = 0.998) mV/decade and detection limits of 1.0 × 10^−6^ and 1.0 × 10^−5^ M. The sampling frequencies were about 22 and 18 samples per hour for sensors (I) and (II), respectively. Typical FI-diagrams for the sensors are shown in Figure 7A,B.

### 3.6. The Analytical Applications

Determination of (ACR^+^) in biological fluids was tested by spiking aliquots of human plasma or urine samples with a known concentration of standard ACR^+^ in 1.0 × 10^−2^ M phosphate buffer of pH 6.0. The results of the added and found ACR are compatible with each other for all sensors with mean recovery between 93–105% for both static and hydrodynamic modes of operation. This confirms the applicability of the method for accurate routine analysis of ACR in biological fluids. In addition, the proposed method was used for ACR^+^ assessment in pharmaceutical sample that currently available in local market (septogel). No interference was caused by active or inactive ingredients and diluents commonly used in the drug formulation. The results obtained on three batches (three determinations each) are shown in Table 5 for static and hydrodynamic mode of operation, respectively.

## 4. Conclusions

Static (manual) and hydrodynamic (FIA) mode of operations techniques are used to determine 9-aminoacridin (ACR) using molecularly imprinted polymers as new receptors in the polymeric membrane sensors. MAA and AM are used as functional monomers in the polymerization process. The simplicity in design, short measurement time, high analytical throughput, low limit of detection and good selectivity are advantages of these sensors. The recognition function of the imprinted polymer membrane towards the ACR is mainly attributed to the size of cavities formed. The sensors are used for determination of ACR^+^ in human biological fluids and septogel drug. No interferences are caused by most ions that normally present in biological fluids or by the active or inactive ingredients and diluents commonly used in drug formulations.

## Figures and Tables

**Table 1:** Scatchard analysis of MIPs and NIPs.

Scatchard Parameters	MIP/MAA/ACR	NIP/MAA/ACR	MIP/AM/ACR	NIP/AM/ACR
K_d1_, μM	1764.91	-	22.14	242.72
Q_max1_, μmol/g	497.35	-	24.37	49.48
K_d2_, μM	401.6	15.04	724.63	487.8
Q_max2_, μmol/g	250.07	35.82	329.71	69.87

**Table 2:** Critical response characteristics of MIP based sensors plasticized with DOP under static mode of operation in 1.0 × 10^−2^ M phosphate buffer of pH 6.0.

Parameters	Sensor (I) MIP/MAA	Sensor (II) MIP/AM	Sensor (III) MIP/MAA/TPB	Sensor (IV) MIP/AM/TPB
Slope, (mV/decade)	51.2 ± 1.3	50.5 ± 1.4	39.9 ± 0.9	41.0 ± 1.6
Correlation coefficient, (r^2^)	0.9997	0.9999	0.9998	0.9998
Linear range, (M)	5.2 × 10^−6^–1.0 × 10^−3^	5.2 × 10^−6^–1.0 × 10^−3^	5.2 × 10^−6^–1.0 × 10^−3^	5.2 × 10^−6^–1.0 × 10^−3^
Detection limit, (M)	2.5 × 10^−7^	8.7 × 10^−7^	1.0 × 10^−6^	8.9 × 10^−7^
Working range, (pH)	4.0–7.0	3.5–7.0	4.0–7.0	3.5–7.0
Response time, (s)	10	10	10	10
Life span, (week)	8	8	8	8
Standard deviation, (mV)	0.98	1.2	1.8	1.6

**Table 3:** Potentiometric selectivity coefficients (log *K^pot^_i,j_*)of ACR membrane sensors plasticized with DOP in 1.0 × 10^−2^ M phosphate buffer of pH 6.0.

Interfering Ion	*Log K^pot^_i,j_*			
	Sensor (I) MIP/MAA	Sensor(II) MIP/AM	Sensor (III) MIP/MAA/TPB	Sensor (IV) MIP/AM/TPB
ACR	0	0	0	0
Piperidine	−2.85	−2.92	−2.88	−2.77
Ethylendiamine	−2.73	−2.86	−2.86	−2.89
3−Aminopyidine	−2.90	−2.93	−3.00	−2.89
Hydroxylamine	−2.89	−2.9	−3.00	−2.92
Histidine	−2.80	−2.94	−3.05	−2.95
Alanine	−2.85	−3.01	−3.07	−2.96
Imidazole	−2.88	−2.95	−3.14	−2.95
Methylamine	−3.04	−3.11	−3.10	−2.98
Hexamine	−2.94	−3.00	−3.05	−3.02
Amprolium HCl	−2.71	−2.86	−3.30	−3.04
Urea	−2.87	−2.98	−3.10	−3.04
Dimethylamine	−2.90	−3.06	−2.86	−3.10

**Table 4:** Performance characteristics of ACR membrane sensors under hydrodynamic mode of operation in 1.0 × 10^−2^ M phosphate buffer of pH 6.0.

Parameters	Sensor (I) MIP/MAA	Sensor (II) MIP/AM
Slope, (mV/decade)	50.1 ± 0.3	50.7 ± 0.2
Correlation coefficient, (r^2^)	0.9988	0.9988
Linear range, M	10^−5^–10^−2^	10^−5^–10^−2^
Detection limit, M	1.0 × 10^−6^	1.0 × 10^−5^
Working range, (pH)	4.0–7.0	3.5–7.0
Response time, (s)	10	10
Life span, (week)	8	8
Flow rate, mL/min	3.5	3.5
Sample rate/h	18	22

**Table 5:** Determination of ACR in spiked plasma, urine and pharmaceutical samples using MIP membrane sensors under static and hydrodynamic modes of operation in 0.01 M phosphate buffer of pH 6.0.

Sample	Added (μM)	Labeled (g)	Static Mode				Hydrodynamic Mode			
			Sensor (I)		Sensor (II)		Sensor (I)		Sensor (II)	
			Found	RSD ^a^	Found	RSD ^a^	Found	RSD ^a^	Found	RSD ^a^
S1 (Urine)	10	-	10.2 ± 0.04	102.0 ± 0.3	-	-	9.78	97.8 ± 0.4	9.77	97.7 ± 0.4
S2 (Urine)	50	-	50.3 ± 0.02	100.6 ± 0.14	51 ± 0.6	102.0 ± 1.2	-	-	-	-
S3 (Urine)	100	-	99.8 ± 0.01	99.8.1 ± 0.7	93.4 ± 0.7	93.4 ± 1.3	93.3	93.3 ± 0.8	95.5	95.5 ± 0.8
S4 (Urine)	1000	-	-	-	906 ± 0.5	90.6 ± 2.0	990	99.0 ± 0.5	1040	104.0 ± 0.5
S5 (Plasma)	10	-	10.2 ± 0.06	102.0 ± 1.3	10.5 ± 1.4	105 ± 2.5	9.64	96.4 ± 0.4	9.77	97.7 ± 0.4
S6 (Plasma)	100	-	94.4 ± 0.05	94.44 ± 2.0	93.3 ± 0.5	93.3 ± 1.2	97.7	97.7 ± 0.4	95.5	95.5 ± 0.4
S7 (Plasma)	1000	-	1090 ± 0.01	109 ± 1.3	934 ± 0.4	93.4 ± 1.5	1050	105.0 ± 0.3	-	-
S8 (Septogel)	-	0.01	0.01058 ± 0.01	105.8 ± 1.2	0.00998 ± 0.05	99.8 ± 1.8	0.00987	98.7 ± 0.1	0.00972	97.2 ± 0.3

^a^ Average of three measurements.
